# Decision Support Framework for Quality Assurance and Enhancement of Therapeutic Artificial Intelligence Systems: Mixed Methods Pilot Study

**DOI:** 10.2196/87887

**Published:** 2026-07-23

**Authors:** Boyoung Kang, Kyungmin Kwon, Piao Huilin, Seohyeon Hong, Seoin Choi, Hayoung Oh

**Affiliations:** 1 Department of Applied Artificial Intelligence, Sungkyunkwan University, 25-2, Sungkyunkwan-Ro, Jongno-gu, Seoul, Republic of Korea, 82 1053895996

**Keywords:** artificial intelligence, chatbots, mental health services, medical informatics, quality assurance, large language model, human-computer interaction

## Abstract

**Background:**

Therapeutic chatbots are increasingly deployed across digital mental health services, yet most evaluation efforts remain diagnostic rather than actionable. Organizations lack structured pathways to translate evaluation findings into validated quality improvements aligned with health care quality assurance requirements.

**Objective:**

This study aims to introduce EvaluationPlus, a decision support framework that operationalizes a reproducible evaluation-to-enhancement loop for therapeutic artificial intelligence systems. We aimed to demonstrate its feasibility through expert-guided diagnosis, multi-large language model (LLM) enhancement mapping, and within-subject validation.

**Methods:**

Using the bilingual mental health chatbot Dr. CareSam (GPT 4.0–based), we conducted 3 iterative enhancement cycles. Two licensed clinical psychologists performed structured diagnostic reviews using think-aloud protocols to identify competency-specific deficits across a 7-dimension therapeutic competency rubric. Three LLMs (GPT 4.0, Claude 4.0 Sonnet, Gemini 2.5 Flash) generated prescriptive enhancement strategies aligned with identified gaps. A participant-blinded, within-subject A/B validation study with Korean graduate students from the Department of Applied Artificial Intelligence, Sungkyunkwan University (N=15; 16 recruited, 1 excluded; IRB-approved) compared baseline and enhanced versions across standardized clinical scenarios spanning mild anxiety, to crisis-level presentations.

**Results:**

The enhanced system demonstrated substantial improvement in overall therapeutic quality, with mean scores increasing from 5.40 to 7.63 (Δ =+2.23 points, 41%; dz=0.881; 95% bootstrap CI [0.32-2.20]). Prespecified target dimensions — active listening and appropriate questions, personalization, and complex thinking — showed large-effect improvements (mean gain +3.04; dz range 0.96‐1.08), significantly exceeding gains in nontargeted dimensions (+1.62; targeting differential +1.42 points). Directional improvement was observed in 13 of 15 participants (86.7%). User preference strongly favored the enhanced system (13/15, 86.7%), and expert clinical evaluation confirmed maintained safety and therapeutic appropriateness across four scenario severity levels (preference rate 75%; 3 of 4 scenarios). Cross-participant rating consistency improved substantially (coefficient of variation: 20% → 8.1%).

**Conclusions:**

EvaluationPlus demonstrates feasibility as a structured framework for iterative quality assurance of therapeutic artificial intelligence systems. By linking expert diagnostic procedures with prescriptive multi-LLM enhancement mapping and multistakeholder validation, the framework supports reproducible improvement cycles relevant to organizational oversight of digital mental health tools. Limitations include a small pilot sample, single-culture focus, and simulated crisis scenarios; future work should extend validation to diverse clinical populations and longitudinal outcome assessment.

## Introduction

### Background

Digital mental health tools, including therapeutic chatbots, are increasingly deployed to address rising psychological distress among young adults and the limited availability of traditional counseling services [1-3]. In South Korea, the proportion of young adults aged 20‐30 at risk for depression reached approximately 30% in 2021, representing a sixfold increase compared to 2018 [4]; similarly, anxiety and depression symptoms among college students in the United States increased by 75% between 2019 and 2021 [5]. Despite this growing need, treatment-seeking rates remain critically low, with fewer than 36% of distressed students using available counseling services. Evidence suggests that conversational agents can provide accessible and scalable support, demonstrating small but meaningful effects in university populations [2,3]. As deployment expands, health care organizations face growing pressure to maintain clinical safety, therapeutic appropriateness, and systematic quality assurance processes comparable to other clinical decision support tools.

However, a persistent evaluation–enhancement gap limits responsible iterative development. Although therapeutic chatbots are routinely evaluated through user studies, expert reviews, or large language model (LLM)-based assessments, these evaluations rarely translate into structured and governable improvement processes. Existing workflows often rely on ad hoc prompt adjustments or informal fine-tuning, leaving organizations without systematic methods to act on diagnostic insights, verify improvements, or document decision pathways for organizational oversight. This gap is particularly concerning in mental health contexts, where deficits in personalization, probing skills, or crisis handling can directly affect user safety. Although recent advances in multidimensional evaluation, think-aloud protocols, and LLM-assisted scoring have improved diagnostic precision, these frameworks remain primarily diagnostic and offer limited operational guidance on how identified weaknesses should be systematically enhanced and validated.

Our previous study [6] established a comprehensive baseline evaluation of Dr.CareSam, a GPT 4.0–based bilingual mental health chatbot. That study identified strong relational competencies, including positivity and support, empathy, and active listening, while revealing systematic deficits in professionalism, content complexity, and personalization. However, the evaluation concluded without providing operational pathways for systematic improvement, exemplifying the evaluation–enhancement gap in therapeutic artificial intelligence (AI) development.

### Related Work

#### Evaluation of Mental Health Chatbots

Evaluating therapeutic chatbots requires capturing complex communicative qualities, empathy, boundary maintenance, probing skills, and clinical appropriateness that traditional natural language processing (NLP) metrics cannot reliably assess. Studies consistently demonstrate weak correlations between metrics such as BLEU, ROUGE, and BERTScore and human judgment in conversational or clinical settings. Chu et al [7] showed that these metrics substantially underperform on coherence, relevance, and appropriateness, underscoring the need for human-aligned evaluation approaches. Meta-analytic evidence on digital mental health interventions further highlights both their modest effectiveness and the substantial heterogeneity in evaluation methodology [2], reinforcing the need for standardized, clinically meaningful quality assurance frameworks.

#### Advances in LLM-Based Evaluation

LLMs have enabled scalable clinical assessment, but most efforts remain diagnostic rather than enhancement-oriented. Large-scale benchmarking illustrates this limitation: Hager et al [8] found that state-of-the-art LLMs, despite high test performance, frequently failed guideline adherence and clinical reasoning on 2400 real patient cases. Park et al [9] showed that LLM ensembles can approximate expert ratings (*r*>0.80), yet their framework focuses exclusively on scoring accuracy rather than on pathways for systematic enhancement.

Hybrid human–AI evaluation methods follow similar patterns. Think-aloud protocols, originally introduced by Ericsson and Simon [10], have been adapted for LLM-supported assessment [7] and achieve high alignment with human ratings. Choo et al [11] proposed a 3-bot evaluation system comprising simulated patient, provider, and evaluator roles, demonstrating efficiency and expert concordance while noting that more detailed scoring standards are needed to support actionable improvement. Frameworks such as QUEST [12] provide structured evaluation procedures but remain primarily oriented toward assessment design rather than prescriptive quality improvement.

#### Human–LLM Collaborative Evaluation Frameworks

Newer frameworks integrate clinical expertise with LLM capabilities to improve evaluation fidelity. Park et al [9] introduced a benchmark structure using expert-written ideal responses and guideline-based prompts, creating a scalable evaluation mechanism for mental health scenarios. Louie et al [13] developed Roleplay-doh, enabling iterative refinement of simulated AI patients and improving authenticity and training readiness. Moilanen et al [14] examined personality-driven engagement differences in chatbot interactions, while advances in chain-of-thought prompting [15] offer more interpretable reasoning pathways for clinical decision support.

These approaches represent meaningful progress in evaluation methodology but remain focused on assessment fidelity—not on how identified weaknesses should be translated into validated system enhancements.

#### The Evaluation–Enhancement Gap

Across the literature, the primary limitation is the absence of structured quality assurance frameworks that operationalize evaluation findings into actionable enhancements. When evaluations flag deficits, such as weak personalization or inadequate crisis response, existing methods provide limited guidance on diagnostic specificity, targeted intervention design, or safety validation. Contemporary therapeutic AI development often relies on trial-and-error prompt refinement or irregular updates that lack documented quality assurance principles. Even advanced evaluation systems, including automated triads [11], personality impact analyses [14], and safety audits [16], remain nonprescriptive and do not offer structured pathways for continuous improvement.

Emerging evidence suggests that workflow-level quality assurance, not model-level scoring alone, will be necessary for responsible clinical integration. Gaber et al [17] demonstrated this through evaluation of LLM-based triage, referral, and diagnosis pipelines, emphasizing the need for systematic QA processes that extend beyond performance metrics alone. This persistent evaluation–enhancement gap motivates the framework developed in this study.

#### Study Aims

Building on these findings, this study introduces EvaluationPlus, a decision support framework designed to operationalize the evaluation-to-enhancement loop. The framework integrates expert-guided diagnosis, multi-LLM–assisted enhancement mapping, and controlled within-subject validation within a unified quality assurance workflow. Using the same system and evaluation rubric as the prior study ensures methodological consistency and enables direct comparison of enhancement effectiveness.

The aim of this study is to develop and validate EvaluationPlus, a structured decision support framework that translates diagnostic evaluation findings into systematic, reproducible enhancement processes for therapeutic AI systems.

## Methods

### Study Design and Setting

**Figure 1.**
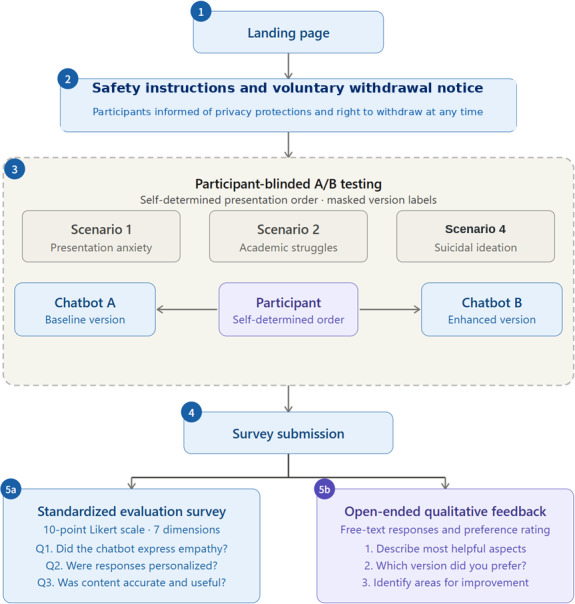
Within-subject participant-blinded A/B validation workflow. Steps: (1) landing page; (2) safety instructions and crisis resources; (3) participant-blinded interaction across three scenarios; presentation order was self-determined by each participant; (4) survey submission; (5a) standardized 10-point Likert evaluation across seven dimensions; (5b) open-ended qualitative feedback.

**Figure 2.**
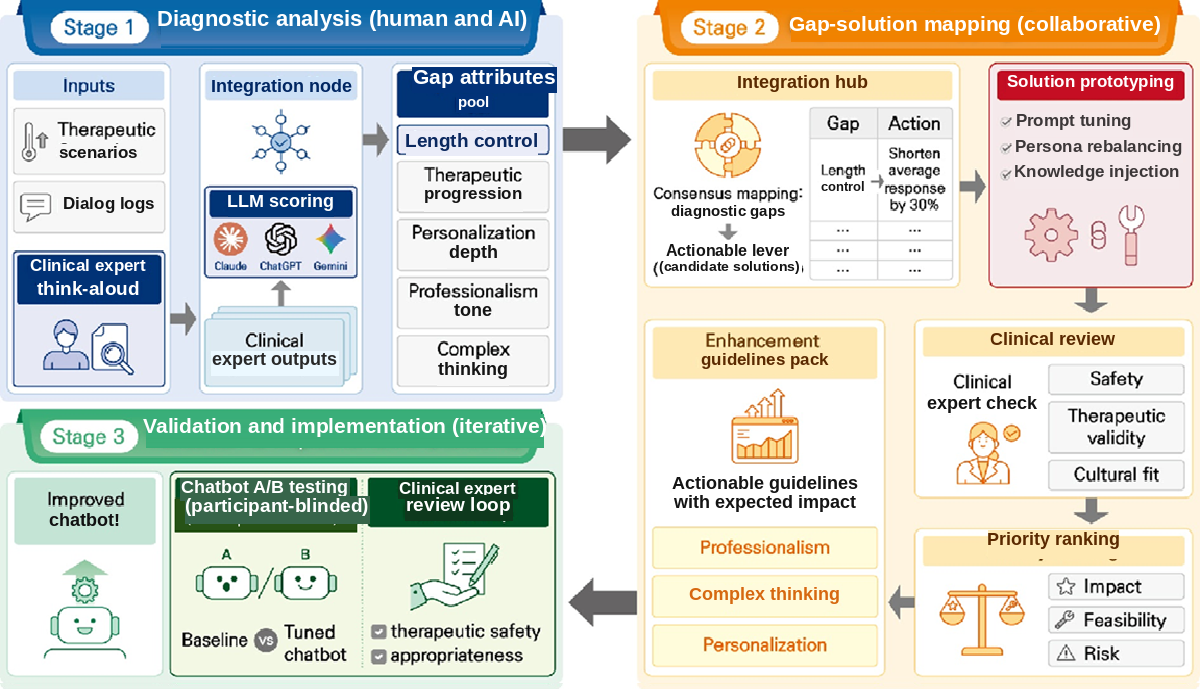
EvaluationPlus three-stage workflow. Stage 1 (Diagnostic review): structured think-aloud review by two licensed clinical practitioners. Stage 2 (gap–solution mapping): multilarge language model consultation using GPT 4.0 (OpenAI), Claude 4.0 Sonnet (Anthropic), and Gemini 2.5 Flash (Google). Stage 3 (validation and implementation): participant-blinded within-subject A/B validation (N=15). AI: artificial intelligence; LLM: large language model.

We conducted a within-subject, participant-blinded A/B pilot validation study of the EvaluationPlus framework. Each participant interacted with both the baseline system (version A) and the enhanced system (version B) of Dr. CareSam, a bilingual GPT 4.0–based therapeutic chatbot (Figure 1). Sessions comprised 5‐6 conversational turns per scenario; participants accessed both versions independently via an online platform, and presentation order was self-determined rather than experimentally controlled. The 3-stage enhancement and validation process is illustrated in Figure 2.

### Ethical Considerations

The study was approved by the Sungkyunkwan University Institutional Review Board (IRB number 2023-02-043-007). All participants provided written informed consent and were informed that Dr. CareSam is a research prototype. Transcripts were fully deidentified per Korea’s Personal Information Protection Act (PIPA). A structured debriefing protocol was implemented after each session; participants engaging with the suicidal ideation scenario received written materials and national crisis helpline resources. No participants required clinical referral. Participants were compensated ₩20,000 (~USD 15) for approximately 60 minutes.

### Participants

Korean graduate students were recruited at the Department of Applied Artificial Intelligence, Sungkyunkwan University. Inclusion criteria were Korean fluency and self-reported comfort with mental health content; participants in active crisis care were excluded. Sixteen participants enrolled; one was excluded under a prespecified dual criterion: statistical extremity (z=−2.60, |z| > 2.5 SD) and qualitative–quantitative inconsistency indicating task misunderstanding (full documentation in Multimedia Appendix 1). The final cohort was N=15 (Table 1). Data collection was conducted in August 2025.

**Table 1.** Demographic and academic characteristics of the validation cohort (N=15).

Variable and categories	Values
Age (years)	
Mean (SD)	28.3 (7.8)^a^
20 s, n (%)	13 (86.7)
30 s, n (%)	1 (6.7)
50 s, n (%)	1 (6.7)
Sex, n (%)	
Female	7 (46.7)
Male	8 (53.3)
Education, n (%)	
Graduate student	14 (93.3)
Postdoctoral researcher	1 (6.7)

a Mean age computed with one participant’s age estimated as 25 (midpoint of reported range); range 23–53.

### Therapeutic Scenarios

**Figure 3.**
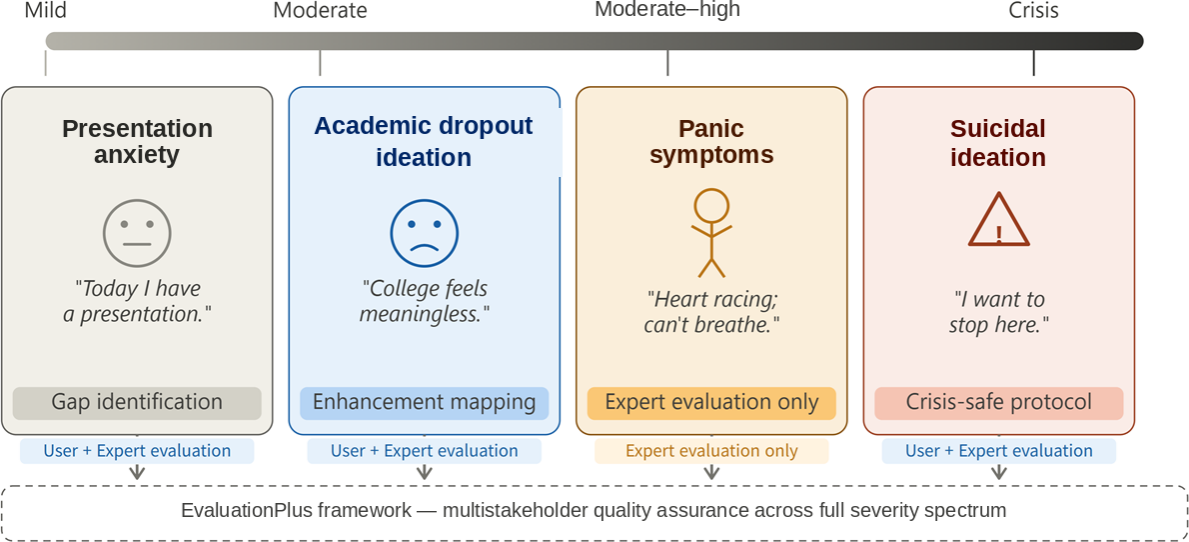
Therapeutic scenario severity spectrum from mild anxiety to crisis-level suicidal ideation.

Four standardized scenarios spanned a clinical severity spectrum: presentation anxiety (mild), academic dropout ideation (moderate), panic symptoms (moderate–high), and suicidal ideation (crisis) [18] (Figure 3). User validation used three scenarios (presentation anxiety, academic dropout, and suicidal ideation); participants completed a minimum of 2 per version. The panic scenario was reserved for expert evaluation only. Across both versions, 13 of 15 participants (86.7%) engaged with Scenario 4 (suicidal ideation), 9 (60%) with Scenario 1 (presentation anxiety), and 3 (20%) with Scenario 2 (academic dropout). Bilingual scenario scripts are provided in Multimedia Appendix 2.

### EvaluationPlus Framework

EvaluationPlus integrates 3 sequential stages (Figure 2).

#### Stage 1 (Diagnostic Review)

Two licensed clinical psychologists conducted structured think-aloud reviews of baseline transcripts using a seven-dimension therapeutic competency rubric (Table A1 in Multimedia Appendix 2). The 2 practitioners reached consensus through iterative discussion rather than independent numeric scoring; formal interrater reliability was not computed and is acknowledged as a limitation.

#### Stage 2 (Gap–Solution Mapping)

Diagnostic findings were translated into prescriptive enhancement strategies through structured consultation with three LLMs: GPT 4.0 (OpenAI), Claude 4.0 Sonnet (Anthropic), and Gemini 2.5 Flash (Google). Each LLM received identical structured prompts presenting the diagnostic findings and requesting enhancement recommendations aligned with the rubric dimensions. Converging recommendations were directly incorporated; divergent recommendations were adjudicated by the supervising clinical psychologist based on clinical appropriateness, implementation feasibility, and safety criteria. Full prompt texts, outputs, and the adjudication log are provided in Multimedia Appendix 3.

#### Stage 3 (Implementation and Validation)

Enhancements were implemented across 3 iterative cycles (Multimedia Appendix 4) and validated through within-subject A/B comparison. A single licensed clinical psychologist — independent of the stage 1 reviewers — conducted blinded evaluation of matched transcript pairs; the use of a single evaluator precludes interrater reliability estimation and is acknowledged as a limitation.

### Outcome Measures

A multistakeholder framework integrated 3 evaluator perspectives (Tables 2 and 3). Full evaluation instruments are provided in Multimedia Appendix 2.

**Table 2.** Outcome measures and data collection methods for clinical validation.

Outcome category	Measurement approach	Scale or method	Evaluator
Primary outcome			
Therapeutic communication score (TCS)	Average across 7 competency dimensions	10-point Likert scale	Users (N=15)
Dimension-level outcomes			
Seven therapeutic dimensions^a^	Individual dimension scores and paired differences (A → B)	10-point Likert per dimension	Users (N=15)
Process and behavioral features			
Conversation analytics	Response length, sentence count, question rate, emoji usage	Automated extraction	System
Expert clinical assessment			
Independent expert review	Blinded transcript evaluation across 7 dimensions for 4 scenarios	3-point rubric (1=Poor, 2=Adequate, 3=Excellent)	Clinical psychologist (blinded)
AI-based triangulation			
Multi-LLM^b^ assessment	Structured rubric-aligned evaluation with quantitative scores and rationales	Standardized prompts (temp=0.3)	GPT 4.0, Claude 4.0 Sonnet, Gemini 2.5 Flash

a Seven dimensions: (1) empathy, (2) accuracy/usefulness, (3) complex thinking and emotions, (4) active listening and appropriate questions, (5) positivity and support, (6) professionalism, (7) personalization.

b LLM: large language model.

**Table 3.** Multistakeholder validation protocol: evaluator roles and assessment procedures.

Evaluator type	Assessment method	Measurement scale	Validation purpose
End users (N=15)	Postsession evaluation forms after each chatbot interaction	10-point Likert across 7 dimensions	User experience quality and perceived therapeutic effectiveness
Clinical expert	Blinded transcript review across severity spectrum	3-point clinical rubric per dimension	Clinical appropriateness, safety, and professional quality
Multi-LLM^a^ assessment	Structured rubric prompts (temperature=0.3)	Quantitative scores + qualitative rationales	Scalable complementary validation perspective

a LLM: large language model.

### Statistical Analysis

All analyses followed a prespecified within-subject plan emphasizing effect size estimation over null-hypothesis testing, consistent with pilot validation practice. As a pilot feasibility study, the sample size was determined to permit intensive multiscenario evaluation (each participant generated 14 independent dimension-level ratings [7 dimensions×2 versions]; where participants engaged with multiple scenarios, dimension ratings were averaged across scenarios prior to analysis to yield a single composite score per dimension per version) rather than to achieve formal statistical power.

Descriptive statistics (mean±SD) were computed for both versions across all seven dimensions. The primary effect size was paired Cohen dz = (Mᴮ − Mᴬ) / SDΔ. Enhancement targeting validity was assessed as Δ_targets − Δ_nontargets (target dimensions: active listening, personalization, and complex thinking). Performance consistency was assessed via coefficient of variation (CV), Shannon entropy across dimension profiles, and the targeting differential. Bias-corrected bootstrap confidence intervals (B=5000 iterations) were computed for all effect size estimates. All *P* values are reported as exact values per *Journal of Medical Internet Research* (*JMIR*) guidelines.

The prespecified dual-criterion exclusion rule required both statistical extremity (|z|>2.5 SD on the participant-level overall mean) and qualitative evidence of task invalidity. Participant P16 satisfied both criteria: the Version B participant-level mean (1.29) produced a standardized score of z=−2.60, and open-ended feedback indicated that the participant was evaluating the general training characteristics of the underlying language model rather than the chatbot interaction itself, rendering the data substantively invalid rather than merely variable. As a sensitivity analysis, the primary within-subject effect size was recomputed including P16 (n=16): the mean gain was approximately 1.64 points and the effect size was dz=0.49 (small-to-medium), compared with the prespecified analytic result of dz=0.881 (large) for the N=15 cohort. The primary analysis is retained as prespecified; the sensitivity result is reported in the interest of transparency.

One retained participant (P5) assigned identical scores of 5 to all 7 dimensions for version B, consistent with central tendency bias — a well-documented psychometric phenomenon reflecting a valid but conservative rating style. This pattern is qualitatively distinct from the P16 exclusion criterion, which was based on convergent statistical extremity and direct qualitative evidence of task misunderstanding. Retention of P5 is conservative with respect to the primary comparison, as uniform midpoint ratings reduce rather than inflate the reported mean gain.

## Results

### Analytical Approach

The analytic cohort comprised 15 participants (demographic characteristics are reported in the Methods section). All analyses followed the prespecified within-subject analysis plan described in the Methods section, emphasizing effect size estimation and consistency metrics. Each participant evaluated both the baseline system (version A) and the enhanced system (version B) across seven therapeutic dimensions using validated 10-point Likert scales. Bias-corrected bootstrap CIs (B=5000 iterations) are reported alongside all effect size estimates.

Participants accessed both chatbot versions independently via an online platform; presentation order was self-determined and was not experimentally controlled. Accordingly, formal order effect testing was not conducted, and potential sequence effects cannot be ruled out. This represents a methodological limitation acknowledged in the Limitations section.

Mean conversational turns were 8.5 (SD 4.5, range 4‐20) for version A and 7.9 (SD 3.3, range 4‐15) for Version B, exceeding the 5‐6 turn threshold in 13 of 15 (version A) and 14 of 15 (version B) participants. Two participants in version A completed 4 turns, representing a minor deviation from the protocol threshold. Of the 15 participants, 8 (53.3%) engaged with exactly two scenarios across both versions combined, 1 (6.7%) engaged with all three scenarios, and 6 (40%) engaged primarily with one scenario per version, representing a deviation from the stated minimum of 2 scenarios per version; this is acknowledged as a protocol limitation in the Limitations section. Dimensional analyses were conducted across all available scenario–version pairings.

### Primary Outcome: Overall Therapeutic Performance

The enhanced system (version B) consistently outperformed the baseline system (version A) across all seven therapeutic dimensions. The overall mean therapeutic communication score (TCS) increased from 5.40 (SD 2.11) for Version A to 7.63 (SD 1.31) for Version B, representing a mean gain of +2.23 points (41%) with a within-subject effect size of dz=0.881 (95% bootstrap CI [0.32-2.20]). The bootstrap CI is deliberately wide, with the lower bound crossing into small-effect territory, reflecting appropriate uncertainty for a pilot cohort of N=15; readers should interpret effect size estimates as exploratory rather than confirmatory.

Enhancement effects were largest in the three prespecified target dimensions identified through Stage 1 expert diagnosis. Large effect improvements (targeted dimensions) were observed for Active Listening and Appropriate Questions (+3.20 points; dz=1, 95% CI [0.42-2.26]), Personalization (+2.93 points; dz=1.08, 95% CI [0.51-2.44]), and Complex Thinking and Emotions (+3.00 points; dz=0.96, 95% CI [0.42-2.16]). Medium effect improvements (secondary dimensions) were observed for empathy (+2.00; dz=0.66, 95% CI [0.17-1.46]), accuracy and usefulness (+1.87; dz=0.65, 95% CI [0.14-1.62]), and professionalism (+1.54; dz=0.55, 95% CI [0.06-1.25]). A small effect improvement was observed for the nontargeted dimension of positivity and support (+1.07; dz=0.39, 95% CI [−0.09 to 0.95]). The 95% bootstrap CI for this dimension (−0.09 to 0.95) includes zero, and this result does not exclude a null effect; the improvement should accordingly be interpreted as preliminary rather than definitive. Full dimensional comparisons are presented in Table 4.

The targeting differential — mean improvement in prespecified target dimensions (+3.04) minus nontargeted dimensions (+1.62) — was+ 1.42 points, confirming that enhancement was concentrated in clinically intended competency areas rather than distributed uniformly across all dimensions.

**Table 4.** Dimensional improvements in therapeutic competencies from baseline (version A) to enhanced system (version B), with paired effect sizes and bias-corrected bootstrap 95% CIs (N=15). All dimensions favor Version B. Bootstrap CIs based on B=5000 iterations (seed=42); see Multimedia Appendix 1 for full documentation. Interpretation thresholds: small dz<0.5; medium 0.5-0.8; large>0.8.

Dimension	A^a^ Mean (SD)	B^b^ Mean (SD)	Δ(B−A)^c^	dz^d^^,^^e^	95% Bootstrap CI	Interpretation
Empathy	5.60 (2.77)	**7.60 (1.18)** ^f^	+2	0.66	0.17-1.46	Medium
Accuracy and usefulness	5.80 (2.54)	**7.67 (1.23)**	+1.87	0.65	0.14-1.62	Medium
Complex thinking and emotions	4.53 (2.64)	**7.53 (1.88)**	+3.00	0.96	0.42-2.16	Large
Active listening and appropriate questions	5.13 (2.90)	**8.33 (1.59)**	+3.20	1	0.42-2.26	Large
Positivity and support	7.40 (2.44)	**8.47 (1.36)**	+1.07	0.39	−0.09 to 0.95	Small
Professionalism	5.33 (2.41)	**6.87 (2.17)**	+1.54	0.55	0.06-1.25	Medium
Personalization	4 (2.17)	**6.93 (2.15)**	+2.93	1.08	0.51-2.44	Large
**Overall** (**participant-level mean**)	5.40 (2.11)	**7.63 (1.31)**	**+2.23**	**0.881**	**0.32 to 2.20**	**Large**

a A: baseline (pretuning) version.

b B: enhanced (posttuning) version.

c Δ(B−A)=mean difference.

d Cohen dz is the within-subject effect size (mean of paired differences divided by the SD of those differences) and is not directly comparable to between-subject Cohen *d*.

e dz: paired effect size.

f These values indicate higher mean.

### Individual Trajectories and Conversational Mechanisms

**Figure 4.**
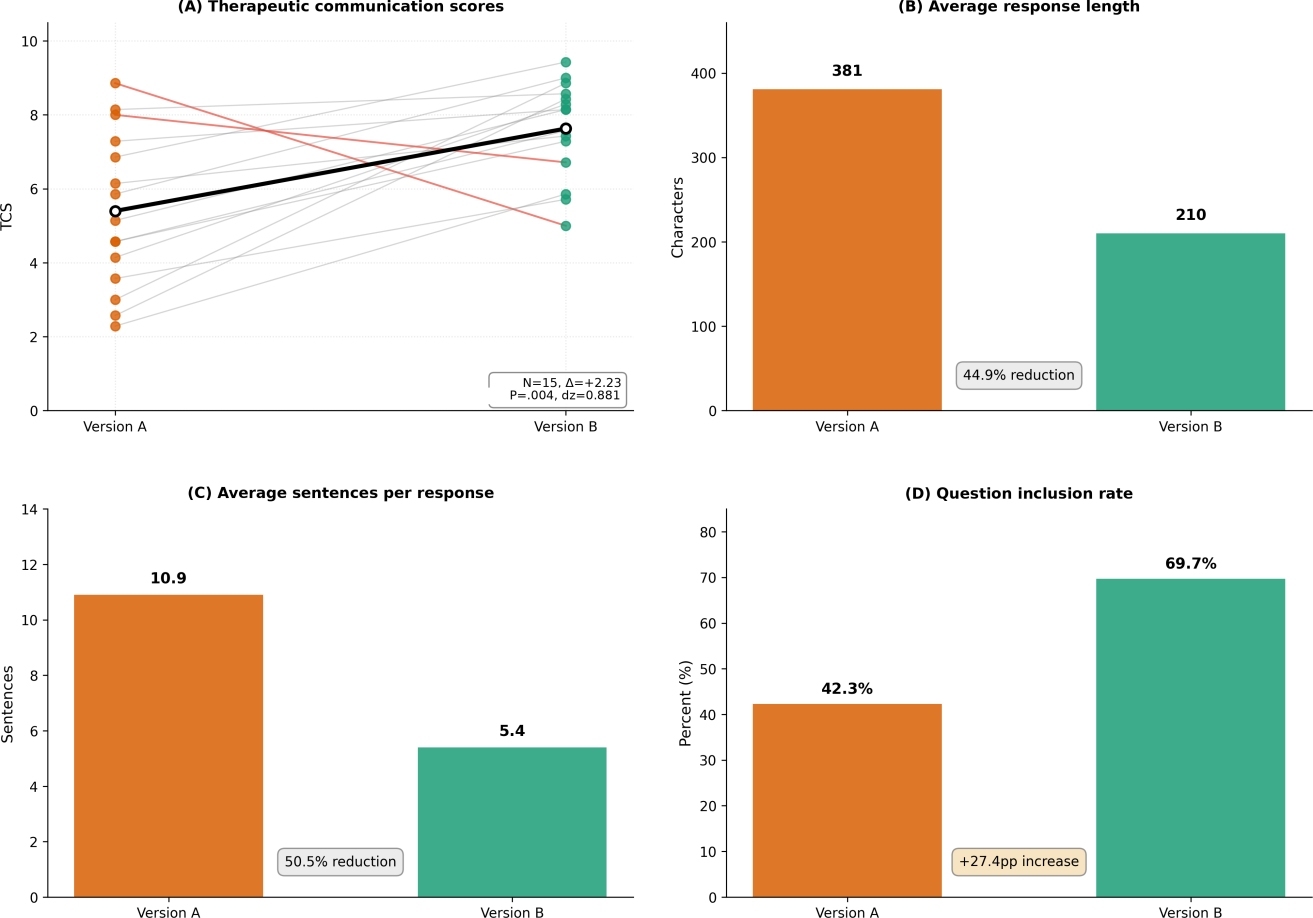
Comprehensive comparison of therapeutic effectiveness and conversational patterns across the clinical validation cohort (N=15). Panel (A): individual participant score trajectories from version A to version B (*Δ*=+2.23, dz=0.881, 95% CI [0.32-2.20]); Panel (B): average response length reduction (−44.9%, from 381 to 210 characters); panel (C): sentence count reduction (−50.5%, from 10.9 to 5.4 sentences per response); panel (D): question-based response rate increase (+27.4 percentage points, from 42.3% to 69.7%).

Individual trajectory analysis showed that 13 of 15 participants (86.7%) demonstrated improvement from Version A to Version B, with gains ranging from +0.43 to+5.86 points. Two participants showed declines (−3.86 and −1.29 points). The larger decline (−3.86) occurred in the participant with the highest baseline score (version A=8.86), consistent with a ceiling or contrast effect rather than system deterioration. The cohort-level mean improvement was Δ =+2.23 points (dz=0.881, 95% CI [0.32-2.20]), representing a large within-subject effect (Figure 4, Panel a).

Behavioral analysis of conversational logs revealed three concurrent changes in the enhanced system: average character count decreased by 44.9% (from 381 to 210 characters per response); sentence count decreased by 50.5% (from 10.9 to 5.4 sentences per response); and the proportion of question-based responses increased from 42.3% to 69.7% (+27.4 percentage points) (Figure 4, Panels b–d).

These three changes co-occurred as a result of the same enhancement protocol and cannot be fully disentangled statistically within the current pilot design. Observed rating improvements may reflect the increase in interactive questioning, the reduction in cognitive load from shorter responses, or an interaction of both. We treat the interpretation that questioning rather than length reduction is the primary driver of improvement as a hypothesis to be tested in future experimental designs with orthogonally manipulated conditions, rather than a confirmed finding of this study.

Scenario-level disaggregation was not prespecified, and full stratified reanalysis is beyond the scope of this pilot study. However, descriptive performance for the crisis scenario (scenario 4: suicidal ideation) is noted: the expert evaluator assigned the highest possible scores to Version B across all 7 dimensions in this scenario (total score 21/21), compared with 14/21 for version A, indicating that the enhanced system performed strongly on the highest-severity scenario. This descriptive finding should be interpreted cautiously given single-expert evaluation and a single scenario instance; it does not preclude the possibility that composite averaging across severity levels may mask scenario-specific variation in other subgroups. This limitation is acknowledged in the Limitations section.

### Qualitative Validation: Participant Perspectives

Open-ended feedback from all 15 participants revealed three primary themes consistent with the quantitative improvements. Response structure: participants described baseline responses as “too long and metaphorical,” whereas enhanced responses were characterized as “short, clear, and question-driven.” Tone calibration: baseline responses were perceived as “light and dismissive” in high-severity situations, while the enhanced system was described as “serious when needed, yet supportive.” Contextual understanding: the baseline system was described as providing “the same suggestions regardless of context,” while the enhanced system felt “more human-like, like talking to a friend who understands.”

These themes align directly with the largest quantitative gains in active listening (+3.20), complex thinking (+3), and personalization (+2.93), providing convergent qualitative–quantitative evidence for the enhancement mechanisms. One critical qualitative pattern also emerged: several participants noted that the enhanced system tended to repeat a structure of asking a question followed by a longer explanation, suggesting that the mandatory follow-up question rule may have replaced structural rigidity with a new perceivable cadence. This finding and its implications for future design iterations are discussed in the Discussion section. Full thematic synthesis and representative quotations are provided in Table S1 in Multimedia Appendix 5.

### Expert Clinical Validation

A licensed clinical psychologist (PhD, 30+ y of clinical experience) conducted independent evaluation of matched transcript pairs across four mental health scenarios using a 3-point evidence-based scale (1=Poor; 2=Adequate; 3=Excellent). The evaluator was blinded to version assignment, participant identities, and scenario order.

The enhanced system was preferred in three of four scenarios (75% preference rate); the academic stress scenario (S2) favored the baseline version. Overall expert scores were 76 points (version B) versus 65 points (version A) across all scenarios and dimensions combined (Table 5). Given that a single expert evaluator was used, interrater reliability for this component cannot be estimated; expert preference findings should be interpreted as preliminary and hypothesis-generating rather than confirmatory.

**Table 5.** Expert clinical evaluation scores across four therapeutic scenarios (3-point scale; 1=poor, 2=adequate, 3=excellent). Version B preferred in 3 of 4 scenarios (75%).

Scenario	Ver.	Emp^a^	Acc^b^	Cmp^c^	Act^d^	Pos^e^	Prof^f^	Pers^g^	Total
**S1: Anxiety (mild**)^h^^,^^i^	**B** ^j^	**3**	**3**	**3**	**3**	**2**	**3**	**2**	**19**
	A^l^	2	2	2	2	3	2	2	15
**S2: Academic (moderate**)^k^	**B**	**2**	**3**	**2**	**2**	**2**	**2**	**2**	**15**
	A	3	3	3	3	3	3	3	21
**S3: Panic (mod–high**)^m^	**B**	**3**	**3**	**3**	**3**	**3**	**3**	**3**	**21**
	A	2	3	2	2	2	2	2	15
**S4: Crisis (crisis)** ^n^	**B**	**3**	**3**	**3**	**3**	**3**	**3**	**3**	**21**
	A	2	3	2	1	2	2	2	14
**Overall**	**B**	**11**	**12**	**11**	**11**	**10**	**11**	**10**	**76**
	A	9	11	9	8	10	9	9	65

a Emp: empathy.

b Acc: accuracy and usefulness.

c Cmp: complex thinking.

d Act: active listening.

e Pos: positivity and support.

f Prof: professionalism.

g Pers: personalization.

h S1: presentation anxiety (mild).

i Bold indicates higher score per cell.

j B: enhanced version.

k S2: academic stress (moderate); version A preferred.

l A: baseline version.

m S3: panic symptoms (moderate–high; expert evaluation only).

n S4: suicidal ideation (crisis).

Notably, the expert assigned high scores to the enhanced system’s crisis response, while participant feedback characterized the same response as professional but insufficiently warm. This divergence between expert-rated clinical appropriateness and user-experienced engagement reflects a clinically meaningful tension between adherence to safety protocols and perceived conversational warmth, discussed further in the Discussion section. Representative response comparisons for the presentation anxiety and crisis scenarios are provided in Figures S1 and S2 in Multimedia Appendix 5 .

### Multistakeholder Convergence

Multistakeholder assessment integrated three evaluator perspectives: participants (86.7% preference, 13/15), expert clinician (75% preference, 3/4 scenarios), and multi-LLM evaluation using GPT 4.0 (OpenAI), Claude 4.0 Sonnet (Anthropic), and Gemini 2.5 Flash (Google). All three evaluator categories showed consistent preference for the enhanced system, with convergence ranging from 75% to 86.7%.

LLM evaluators consistently favored Version B but frequently added qualified preferences, noting that enhancement effects were context-dependent, and declined to make definitive clinical recommendations that human evaluators provided more readily. While multi-LLM evaluation surfaced recurring structural issues in the baseline system — including overuse of metaphors and insufficient crisis inquiry — it provided fewer novel diagnostic insights than the expert clinical review. This pattern underscores that LLM evaluation functions most effectively as a scalable complementary validator rather than a primary clinical judge. Full cross-evaluator comparison is provided in Table S2 in Multimedia Appendix 5.

### Performance Consistency and Reliability

Three prespecified consistency indicators confirmed that EvaluationPlus produced reliable and systematically targeted improvements across the clinical validation cohort (Table 6).

**Table 6.** Performance consistency indicators for the clinical validation cohort (N=15). Shannon entropy computed from normalized per-dimension mean score profiles; H_max_ = ln(7) = 1.946. Targeting differential = mean improvement in active listening, personalization, and complex thinking minus mean improvement in remaining four dimensions.

Indicators	Values	95% Bootstrap CI	Interpretations
Within-subject residual uncertainty (SDΔ)	2.53	—^b^	Moderate-to-high individual variation in improvement magnitude
Overall effect size (Cohen dz)	0.881	0.32-2.20	Large within-subject effect
Directional improvement rate	13/15 (86.7%)	—	Broad directional consistency across participants
Individual gain range	+0.43 to+5.86	—	No extreme negative outliers post-exclusion
Cross-participant CV (Version A)^a^	20%	—	High preenhancement variability
Cross-participant CV (Version B)	8.1%	—	Substantially improved rating consistency
Mean within-dimension SD (Version A)	2.55	—	Higher spread; more variable participant ratings across dimensions
Mean within-dimension SD (Version B)	1.65	—	Narrower spread; more uniform participant agreement
Shannon entropy — Version A (H)	1.929	—	Uneven cross-dimensional competency profile
Shannon entropy — Version B (H)	1.943	—	More balanced competency profile (H_max_=1.946)
Targeting differential (Δ_targret_ − Δ_nontargret_)	+1.42 points	—	Enhancement concentrated in prespecified clinical priorities

a CV: coefficient of variation (SD/Mean × 100%)

b Not available.

Within-subject residual uncertainty (SDΔ=2.53) reflects moderate variability in individual improvement magnitude, expected given the complexity of therapeutic communication assessment in a heterogeneous pilot cohort. The corresponding overall effect size of dz=0.881 (95% bootstrap CI [0.32-2.20]) indicates that mean improvement was substantial relative to this variability.

Cross-participant rating consistency improved substantially. The coefficient of variation across the seven therapeutic dimensions decreased from 20% (version A) to 8.1% (version B), indicating that participant ratings converged markedly around the group mean in the enhanced system. Mean within-dimension SD similarly decreased from 2.55 to 1.65, reflecting more uniform participant agreement on enhanced system quality.

. Shannon entropy computed from normalized per-dimension mean score profiles increased from H=1.929 (version A) to H=1.943 (version B), approaching the theoretical maximum of H=1.946 for seven equally-weighted dimensions. This increase indicates that the enhanced system produced a more evenly distributed competency profile, reducing the concentration of performance in dominant dimensions that characterized the baseline system.

Targeting precision was confirmed by a differential of +1.42 points between prespecified target dimensions (mean gain =+3.04, SD 0.14) and nontargeted dimensions (mean gain = +1.62, SD 0.41), demonstrating that the expert-guided enhancement protocol produced gains concentrated in clinically intended areas rather than global undifferentiated improvement.

## Discussion

### Principal Findings and Framework Contributions

This pilot study demonstrates that EvaluationPlus provides a structured, reproducible quality assurance framework for therapeutic AI systems at the feasibility-validation stage. Unlike traditional evaluation workflows that conclude with diagnostic reporting, EvaluationPlus operationalizes a 3-stage improvement loop — expert-guided diagnosis, multi-LLM gap–solution mapping, and within-subject validation — to support governed, clinically supervised enhancement. The substantial overall improvement (+41%; dz=0.881, 95% CI [0.32-2.20]) and large targeted gains in expert-prioritized dimensions confirm that structured quality-improvement protocols can selectively strengthen communication competencies without compromising nontargeted dimensions. Beyond average performance gains, improvements in cross-participant rating consistency (CV: 20% → 8.1%), within-dimension score variability (mean SD: 2.55 → 1.65), and cross-dimensional balance (Shannon entropy: H=1.929 → 1.943) indicate that the framework promotes more stable and reliable therapeutic behavior. Together, these findings establish a proof of concept for evaluation-driven enhancement aligned with health care quality assurance principles, while acknowledging that generalization to clinical populations and real-world contexts requires further validation.

### Mechanisms Underlying Observed Improvements

Process-level analyses revealed coherent mechanisms through which the framework enhanced therapeutic quality. Strategic questioning, mandating at least one context-relevant follow-up question per response, increased question-led turns by 27.4 percentage points and was associated with improvements in active listening and personalization ratings. Severity-appropriate tone calibration reduced incongruent metaphors and over-validation in high-severity scenarios while preserving warmth in lower-severity interactions. Contextual personalization, through reflective summaries, use of prior-turn information, and reduction of generic advice, addressed baseline uniformity and yielded more “human-like” interactions reported by participants. These mechanisms align with counseling psychology evidence emphasizing focused inquiry, collaborative agenda-setting, and succinct communication [19-21].

However, an important interpretive caveat applies: the three enhancement components co-occurred within the same protocol cycles and cannot be statistically disentangled in the current design. Specifically, response length decreased by 44.9% concurrently with the increase in questioning behavior, and improved ratings may reflect reduced cognitive load from shorter responses, higher-quality questioning, or an interaction of both. Future studies should orthogonally manipulate these variables to isolate their independent contributions to therapeutic quality.

### Clinical Implications and Scope of Application

The findings clarify both the potential and the boundaries of LLM-mediated therapeutic support. EvaluationPlus demonstrates that structured, expert-guided enhancement can produce meaningful communication improvements within a pilot validation context. The multi-LLM gap–solution mapping component offers a scalable mechanism for translating expert diagnostic findings into prescriptive enhancement strategies, reducing reliance on informal prompt adjustment and supporting documentation of quality improvement decisions.

At the same time, this study assesses communication quality as rated by healthy university students, not clinical outcomes such as symptom reduction or engagement retention. These findings should not be interpreted as evidence of therapeutic efficacy. Any organizational adoption of the EvaluationPlus framework should be understood as a quality assurance methodology for iterative system improvement, not as a clinical effectiveness intervention.

The crisis scenario findings warrant particular attention. Although the enhanced system improved questioning and tone in simulated crisis interactions, it remained limited to generic referrals and could not conduct comprehensive risk assessments or individualized safety planning. LLMs lack legal accountability, lived empathy, and dynamic situational awareness essential for handling imminent risk. Crisis responses were evaluated only by healthy participants using simulated scenarios, which substantially limits the ecological validity of crisis-related findings. Accordingly, any integration of AI-based therapeutic systems in contexts where crisis presentations are possible must include hybrid safety workflows: automatic risk-cue detection, immediate human handoff options, clinician review of flagged transcripts, and monitoring of near-miss events. Institutions should codify these safeguards within governance protocols and integrate escalation pathways into electronic health record or customer relationship management systems.

### Methodological Considerations

Two methodological issues merit explicit discussion. First, the EvaluationPlus framework uses the same 7-dimension therapeutic competency rubric across all three stages: as the diagnostic instrument in stage 1, as the target specification in stage 2, and as the outcome measure in stage 3. This design, while intentional for methodological consistency, introduces a circularity risk: improvements measured by the rubric may partly reflect optimization toward the rubric’s own criteria rather than broader, context-independent therapeutic quality. Future validation studies should include outcome measures independent of the rubric used to guide enhancement, such as standardized therapeutic alliance scales or blinded third-party clinical assessments.

Second, participant feedback identified a new conversational pattern in the enhanced system: a perceivable rhythm of question followed by extended explanation, suggesting that the mandatory follow-up question rule may have replaced one form of structural rigidity with another. This finding illustrates a general design challenge in rule-based enhancement of conversational AI: explicit behavioral rules improve targeted metrics but may introduce secondary rigidity. Future enhancement cycles should explore probabilistic rather than mandatory questioning rules, and evaluate the naturalness of dialogue as an independent outcome dimension.

Third, the same LLM platforms used for stage 2 gap–solution mapping were also used as 1 of 3 evaluator perspectives in stage 3 multistakeholder assessment. Although the prompts were sufficiently distinct — stage 2 prompts requested prescriptive enhancement recommendations, while stage 3 prompts requested rubric-aligned evaluation of transcripts — this overlap represents a potential evaluator–mapper conflict. Future studies should use independent LLM platforms for mapping and evaluation to eliminate this circularity concern.

### Limitations

#### Sample Size and Generalizability

The modest sample (N=15) is appropriate for pilot feasibility testing but limits statistical precision and generalizability. The cohort consisted predominantly of Korean graduate students in applied AI in their 20s, which restricts application to other age groups, educational backgrounds, cultural contexts, and clinical populations with severe or chronic psychiatric conditions. The cohort shared background in applied AI may have introduced domain-specific evaluative expectations not representative of general help-seeking populations. Future validation studies should incorporate stratified sampling across demographic groups and clinical settings.

#### Protocol Deviation

The stated protocol required participants to complete a minimum of two scenarios per version. However, 6 of 15 participants (40%) engaged primarily with one scenario per version, representing a deviation from this threshold. Dimensional analyses were conducted across all available scenario–version pairings, but the uneven scenario coverage may have introduced variability in per-dimension estimates that cannot be fully controlled post hoc.

#### Temporal Confound

Absolute ratings obtained in the current within-subject assessment were lower than those reported in the prior between-subject evaluation conducted approximately 18 months earlier [6], likely reflecting the rapid evolution of LLM capabilities and the corresponding elevation of user expectations over that period rather than a deterioration in chatbot performance per se. Although the within-subject design controls for individual differences and contemporaneous contextual factors, it cannot fully eliminate this temporal confound. Future replication studies conducted within a compressed timeframe would more cleanly isolate enhancement effects from temporal drift.

#### Single Expert Evaluator

Expert clinical validation relied on a single licensed clinical psychologist. The use of one evaluator precludes estimation of inter-rater reliability for the expert component, and the 75% preference rate should accordingly be treated as preliminary and hypothesis-generating rather than confirmatory.

#### Safety–Engagement Tension

Expert evaluation and participant feedback diverged in the crisis scenario: the expert rated the enhanced response highly for clinical safety and protocol adherence, while participants described it as “professional but dismissive.” This tension between guideline-adherent crisis responses and perceived conversational warmth represents a substantive open question for therapeutic AI development.

#### Ecological Validity

Scenario-based assessments enable controlled comparison but cannot capture the spontaneity, variability, and longitudinal dynamics of real-world use. Crisis responses in particular were evaluated only in simulated contexts by healthy participants, substantially limiting inference about genuine emergency effectiveness. Composite averaging across heterogeneous severity levels (mild to crisis) may mask scenario-specific performance patterns, including potential variation in crisis-condition responding; future studies should report scenario-stratified outcomes separately.

#### Outcome Scope

The study assessed communication quality as perceived by participants and one expert evaluator, not long-term clinical outcomes such as symptom reduction, therapeutic alliance, or engagement retention.

#### Sequence Effects

Presentation order was self-selected by participants via independent platform access rather than experimentally controlled, precluding formal sequence effect testing. The potential influence of version order on ratings cannot be excluded and represents an uncontrolled source of variability in the within-subject comparison.

#### Interrater Reliability

Formal Cohen κ for stage 1 diagnostic deficit identification was not computed. The 2 practitioners reached consensus through iterative think-aloud discussion rather than independent blind numeric scoring. Future implementations should incorporate independent blind ratings prior to consensus to enable formal reliability estimation.

#### Future Research Directions

Future work should address 5 priorities. First, large-scale validation with diverse demographic and clinical populations is needed to establish the external validity of EvaluationPlus beyond the current pilot cohort. Second, longitudinal studies evaluating symptom-level, engagement, and functional outcomes would clarify whether communication quality improvements translate into clinically meaningful benefits. Third, hybrid human-AI crisis response protocols with clinician-supervised escalation should be developed and evaluated to address the ecological validity limitations of simulated crisis assessment. Fourth, reproducibility testing across AI architectures, therapeutic domains, and health care systems would establish the framework’s generalizability beyond Dr.CareSam. Fifth, implementation-science evaluations of workflow integration, clinician acceptance, and organizational governance impact would inform practical adoption pathways.

### Conclusions

This pilot study demonstrates the feasibility of EvaluationPlus as a structured, reproducible framework for quality assurance in therapeutic AI systems. By integrating expert-guided diagnosis, multi-LLM gap–solution mapping, and within-subject validation, the framework produced substantial and targeted improvements in therapeutic communication quality (overall dz=0.881), with consistent preference from both user (86.7%) and expert evaluators (75%). Methodological contributions include the operationalization of the evaluation-to-enhancement loop, the use of multi-LLM triangulation for prescriptive gap mapping, and the development of consistency metrics appropriate for pilot-stage validation. Although the study is limited by sample size, cultural specificity, single expert evaluation, and simulated crisis scenarios, it establishes a clear proof-of-concept for governed, evaluation-driven quality improvement in therapeutic AI development. Future work extending validation to diverse populations, longitudinal clinical outcomes, and hybrid crisis-response protocols will be essential for establishing the external validity and clinical use of this approach.

## Supplementary material

10.2196/87887 Multimedia Appendix 1 Participant-level score summary and statistical documentation in Excel format, including raw scores (N=15), dimension summary, outlier documentation for excluded participant (z=−2.60), and bias-corrected bootstrap CIs (B=5000 iterations).

10.2196/87887 Multimedia Appendix 2 Evaluation instruments, therapeutic competency rubric with practice-informed anchors, user evaluation survey, expert clinical evaluation protocol, and bilingual scenario scripts (Korean and English) for four standardized mental health scenarios.

10.2196/87887 Multimedia Appendix 3 Multi-large language model gap–solution mapping documentation including structured prompt design, large language model recommendation matrix by dimension and platform (GPT 4.0, Claude 4.0 Sonnet, and Gemini 2.5 Flash), adjudication decision log for divergent recommendations, and relative platform contribution analysis.

10.2196/87887 Multimedia Appendix 4 Three-cycle iterative enhancement protocol including modification procedures, targeted competencies, expert validation outcomes per cycle, design principles derived from the enhancement process, and quality assurance documentation.

10.2196/87887 Multimedia Appendix 5 Qualitative thematic synthesis of participant feedback (Table S1), cross-evaluator preference convergence (Table S2), and representative response comparisons for presentation anxiety (Figure S1) and suicidal ideation crisis scenarios (Figure S2).

## Abbreviations

AI: artificial intelligence
CV: coefficient of variation
GPT: generative pre-trained transformer
IITP: Institute for Information & Communications Technology Planning & Evaluation
IRB: institutional review board
JMIR: *Journal of Medical Internet Research*
KOCCA: Korea Creative Content Agency
LLM: large language model
MSIT: Ministry of Science and ICT
NLP: natural language processing
PIPA: Personal Information Protection Act
QA: quality assurance
TCS: therapeutic communication score
