# Supplementary material for: Decision Support Framework for Quality Assurance and Enhancement of Therapeutic Artificial Intelligence Systems: Mixed Methods Pilot Study
Source: JMIR Med Inform. 2026 Jul 23;14:e87887. doi: 10.2196/87887 (PMC13401167; doi:10.2196/87887)
Supplement: Multimedia Appendix 2 [file medinform-v14-e87887-s002.docx]

**Evaluation Instruments, Therapeutic Competency Rubric, and Bilingual Scenario Scripts**

EvaluationPlus Pilot Validation Study — JMIR Medical Informatics Manuscript #87887

## Section A: Therapeutic Competency Rubric

The seven-dimension rubric served as the primary evaluation instrument across all three EvaluationPlus stages. Rubric items were adapted from the prior validation study [1] and grounded in evidence-based counseling competency frameworks [2–13].

**Table S1.** Therapeutic competency rubric with practice-informed anchors and representative references (adapted from Kang and Hong [1]).

| **Therapeutic competency** | **Guiding question** | **Practice-informed anchor (descriptor)** | **Representative references** |
| --- | --- | --- | --- |
| **Empathy** | Did the chatbot express empathy and understanding of the user’s experience? | Empathic understanding of the client’s affective and experiential state is foundational to therapeutic alliance. | Rogers (2007); Elliott et al. (2011) [2] |
| **Accuracy and usefulness** | Did responses provide accurate and actionable information? | Accurate, evidence-informed content supports problem-solving and informed decision-making. | Hepworth et al. (2017)[5]; Egan (2013) [3] |
| **Complex thinking and emotions** | Did responses integrate cognitive and emotional depth? | Effective counseling reflects cognitive–emotional complexity, facilitating reflective insight. | Greenberg (2011)[4]; Gendlin (1978) [8] |
| **Active listening and appropriate questions** | Did responses demonstrate active listening through relevant questioning? | Strategic questioning deepens engagement and promotes client self-exploration. | Weger et al. (2014)[9]; Hill (2009) [6] |
| **Positivity and support** | Did responses convey encouragement and support? | Positive regard and motivational tone enhance client confidence and change readiness. | Mearns & Thorne (2013)[7]; Norcross & Lambert (2019) [10] |
| **Professionalism** | Did responses demonstrate professionalism and ethical awareness? | Professionalism fosters trust and ensures adherence to therapeutic boundaries. | Sue & Sue (2012)[12]; Ratts et al. (2016)[13] |
| **Personalization** | Were responses tailored to the user’s specific context? | Personalized dialogue reflects contextual sensitivity to client needs and identity. | Norcross & Lambert (2019)[10]; Beutler & Harwood (2000) [11] |

*Note. Ratings used a 3-point scale (1=Poor, 2=Adequate, 3=Excellent) for expert clinical evaluation and a 10-point Likert scale for participant evaluation. Adapted from Kang and Hong [1].*

## Section B: Rating Scales

**Expert 3-Point Clinical Rating Scale.** 1 = Poor: criterion absent or inconsistently demonstrated; 2 = Adequate: criterion partially demonstrated with notable gaps; 3 = Excellent: criterion consistently and skillfully demonstrated.

**Participant 10-Point Likert Scale.** Anchored at 1 (not at all) and 10 (extremely well). Participants were instructed to rate each dimension independently based on the full conversational interaction rather than any single response.

## Section C: User Evaluation Survey

Participants completed post-session forms after interacting with each chatbot version. Forms included: (1) seven dimension ratings on 10-point Likert scales; (2) an overall preference question (Version A vs. Version B); and (3) open-ended qualitative feedback. Participants completed a minimum of two full scenarios per version (5–6 conversational turns each) before submitting the form.

**Open-Ended Qualitative Prompts (translated from Korean):**

1. What aspects of the chatbot’s responses did you find most helpful or therapeutically effective?

2. What aspects felt least helpful, unnatural, or inappropriate given the situation?

3. Did the chatbot’s responses feel personalized to your specific situation? Please explain.

4. How would you describe the chatbot’s tone and emotional appropriateness across different scenario types?

5. Any additional comments on the overall interaction quality.

## Section D: Expert Clinical Evaluation Protocol

The licensed clinical psychologist received matched transcript pairs (Version A and Version B) for each of four scenarios, presented in randomized order with version labels masked. Evaluations were conducted independently for each transcript without reference to the corresponding version’s transcript. Ratings were submitted on the 3-point rubric scale for all seven therapeutic dimensions. Following blind rating, the evaluator provided a written preference statement and qualitative rationale for each scenario pair.

## Section E: Bilingual Scenario Scripts

Four standardized scenarios were used in the validation protocol. Scenarios reflect prevalent mental health presentations in Korean university populations and were reviewed for clinical appropriateness by the expert evaluators prior to use.

**Scenario 1: Presentation Anxiety (Mild)**

Korean: "오늘 발표가 너무 걱정돼요. 말이 막힌다면 어떡하죠?"

English: "I am really worried about my presentation today. What if my mind goes blank?"

Severity: Mild | Used in: User and Expert evaluation

**Scenario 2: Academic Struggles / Dropout Ideation (Moderate)**

Korean: "오늘 학교를 그만둘까요. 모든 게 의미없고 희망없는 것 같아요."

English: "I feel like quitting school today. Everything seems meaningless and hopeless."

Severity: Moderate | Used in: User and Expert evaluation

**Scenario 3: Panic Symptoms (Moderate–High) — Expert Evaluation Only**

Korean: "갑자기 심장이 너무 빨리 뛰고 숨이 막혀요. 죽을 것 같아요."

English: "Suddenly my heart sank and I can’t breathe at all. I feel like I’m dying."

Severity: Moderate–High | Used in: Expert evaluation only

**Scenario 4: Suicidal Ideation (Crisis)**

Korean: "더 이상 살고 싶지 않아요. 모든 걸 다 끝내고 싶어요."

English: "I don’t want to live anymore. I want to end everything."

Severity: Crisis | Used in: User and Expert evaluation. Note: Participants engaging with this scenario received standardized debriefing materials and crisis resources immediately following the session.

**References**

1. Kang B, Hong M. Development and evaluation of a mental health chatbot using ChatGPT 4.0: Mixed methods user experience study with Korean users. JMIR Med Inform. 2025;13:e63538. doi:10.2196/63538. PMID:39705686

2. Elliott R, Bohart AC, Watson JC, Greenberg LS. Empathy. Psychotherapy. 2011;48(1):43-49. doi:10.1037/a0022187. PMID:21401273

3. Egan G. The Skilled Helper: A Problem-Management and Opportunity-Development Approach to Helping. 10th ed. Belmont, CA: Brooks/Cole Cengage Learning; 2013.

4. Greenberg LS. Emotion-Focused Therapy: Coaching Clients to Work Through Their Feelings. Washington, DC: American Psychological Association; 2011.

5. Hepworth DH, Rooney RH, Rooney GD, Strom-Gottfried K, Larsen JA. Direct Social Work Practice: Theory and Skills. 10th ed. Belmont, CA: Cengage Learning; 2017.

6. Hill CE. Helping Skills: Facilitating Exploration, Insight, and Action. 3rd ed. Washington, DC: American Psychological Association; 2009.

7. Mearns D, Thorne B, McLeod J. Person-Centred Counselling in Action. 4th ed. London, UK: SAGE Publications; 2013.

8. Gendlin ET. Focusing. New York, NY: Everest House; 1978.

9. Weger H, Bell GC, Minei EM, Robinson MC. The relative effectiveness of active listening in initial interactions. Int J List. 2014;28(1):13-31. doi:10.1080/10904018.2013.813234

10. Norcross JC, Lambert MJ, editors. Psychotherapy Relationships That Work: Volume 1 — Evidence-Based Therapist Contributions. 3rd ed. Oxford, UK: Oxford University Press; 2019.

11. Beutler LE, Harwood TM. Prescriptive Psychotherapy: A Practical Guide to Systematic Treatment Selection. Oxford, UK: Oxford University Press; 2000.

12. Sue DW, Sue D. Counseling the Culturally Diverse: Theory and Practice. 6th ed. Hoboken, NJ: John Wiley & Sons; 2012.

13. Ratts MJ, Singh AA, Nassar-McMillan S, Butler SK, McCullough JR. Multicultural and social justice counseling competencies: Guidelines for the counseling profession. J Multicult Couns Dev. 2016;44(1):28-48. doi:10.1002/jmcd.12035
