# Supplementary material for: Decision Support Framework for Quality Assurance and Enhancement of Therapeutic Artificial Intelligence Systems: Mixed Methods Pilot Study
Source: JMIR Med Inform. 2026 Jul 23;14:e87887. doi: 10.2196/87887 (PMC13401167; doi:10.2196/87887)
Supplement: Multimedia Appendix 3 [file medinform-v14-e87887-s003.docx]

**Multi-LLM Gap–Solution Mapping — Prompt Strategy, Platform Outputs, and Adjudication Process**

EvaluationPlus Pilot Validation Study — JMIR Medical Informatics Manuscript #87887

## Section A: Prompt Design

Each of the three LLM platforms (GPT 4.0, Claude 4.0 Sonnet, Gemini 2.5 Flash) received an identical structured system prompt comprising three components: (1) the seven-dimension therapeutic competency rubric (Multimedia Appendix 1); (2) a summary of Stage 1 diagnostic findings for each dimension, presented as standardized deficit statements; and (3) a structured output request specifying the format for enhancement recommendations (dimension, target behavior, implementation rule, priority level).

**Example diagnostic deficit statement format:** “Dimension: Active Listening and Appropriate Questions — Deficit: Responses do not include follow-up questions relevant to the user’s specific situation; chatbot provides statements without eliciting user engagement or narrative elaboration.”

**Structured output format requested from each LLM:**

Dimension: [name] | Priority: [High/Medium/Low] | Target behavior: [specific observable behavior] | Implementation rule: [concrete instruction for prompt modification] | Rationale: [clinical or communicative justification]

## Section B: LLM Recommendation Matrix

Table S1 summarizes enhancement recommendations generated by each platform across the seven therapeutic dimensions. Convergence status indicates whether all three platforms independently generated equivalent recommendations (Converged) or partial agreement was reached (Partial).

**Table S1.** Enhancement recommendation matrix by therapeutic dimension and LLM platform (Stage 2 Gap–Solution Mapping).

| **Dimension** | **GPT 4.0** | **Claude 4.0 Sonnet** | **Gemini 2.5 Flash** | **Convergence** |
| --- | --- | --- | --- | --- |
| **Empathy** | **Reduce extended emotional validation; use 1–2 reflective statements** | **Increase use of person-centered language; mirror emotional vocabulary** | **Strengthen empathic phrasing; reduce generic expressions** | **Converged** |
| **Accuracy and usefulness** | **Add concrete actionable steps to each response** | **Prioritize evidence-based suggestions over motivational content** | **Include specific resource recommendations** | **Converged** |
| **Complex thinking and emotions** | **Integrate cognitive reframing explicitly** | **Connect emotional and cognitive content within each response** | **Deepen reasoning chains; avoid surface-level validation** | **Converged** |
| **Active listening and appropriate questions** | **Mandate ≥1 context-specific follow-up question per response** | **Increase open-ended questioning frequency** | **Replace rhetorical statements with genuine inquiry** | **Converged** |
| **Positivity and support** | **Calibrate positivity to severity; reduce in crisis contexts** | **Maintain encouragement; adjust tone not content** | **Preserve positive framing; modulate intensity** | **Partial** |
| **Professionalism** | **Enforce emoji prohibition in high-severity scenarios; four-stage structure** | **Adopt formal tone for clinical content; avoid colloquial language** | **Strengthen boundary language; reference professional services explicitly** | **Converged** |
| **Personalization** | **Replace generic advice with reflective summaries using prior-turn content** | **Reference specific user statements; avoid template responses** | **Incorporate user-specific details into each response** | **Converged** |

*Note. GPT-4.0 = OpenAI GPT 4.0; Claude 4.0 Sonnet = Anthropic Claude 4.0 Sonnet; Gemini 2.5 Flash= Google Gemini 2.5 Flash. All platforms used temperature=0.3 for consistency. Converged = all three platforms generated equivalent recommendations; Partial = two of three platforms agreed.*

## Section C: Adjudication of Divergent Recommendations

For the single dimension with partial convergence (Positivity and Support), the supervising clinical psychologist reviewed all platform outputs and made a binding implementation decision. Table S2 documents the adjudication process.

**Table S2.** Adjudication decision log for partially converging LLM recommendations (Positivity and Support dimension).

| **Dimension** | **Divergent Recommendations** | **Clinical Rationale for Adjudication** | **Final Implementation Decision** |
| --- | --- | --- | --- |
| **Positivity and support** | **GPT 4.0 recommended calibrating positivity to severity; Claude and Gemini recommended preserving positive framing throughout** | **Clinical appropriateness: excessive positivity in crisis contexts may reduce perceived safety and deter help-seeking** | **GPT 4.0 recommendation adopted; severity-adaptive positivity modulation implemented in Cycle 2** |
| **Dimension** | **Divergent Recommendations** | **Clinical Rationale** | **Final Implementation Decision** |

*Note. Adjudication conducted by senior licensed clinical psychologist (30+ years experience). Decision finalized prior to Cycle 2 implementation.*

## Section D: Relative Platform Contributions

GPT 4.0 demonstrated strongest performance on structural scaffolding recommendations (e.g., turn-sequencing, response length targets). Claude 4.0 Sonnet generated more nuanced recommendations for empathic language and reflective questioning patterns, consistent with evidence emphasizing focused inquiry and collaborative agenda-setting [2,3]. Gemini 2.5 Flash contributed most distinctively to crisis-tier protocol recommendations, particularly in specifying escalation language and resource referral formats [4,5]. These complementary strengths informed the decision to use a three-platform ensemble for the Gap–Solution Mapping stage.

**References**

1. Kang B, Hong M. Development and evaluation of a mental health chatbot using ChatGPT 4.0: Mixed methods user experience study with Korean users. JMIR Med Inform. 2025;13:e63538. doi:10.2196/63538. PMID:39705686

2. Elliott R, Bohart AC, Watson JC, Greenberg LS. Empathy. Psychotherapy. 2011;48(1):43-49. doi:10.1037/a0022187. PMID:21401273

3. Hill CE. Helping Skills: Facilitating Exploration, Insight, and Action. 3rd ed. Washington, DC: American Psychological Association; 2009.

4. Weger H, Bell GC, Minei EM, Robinson MC. The relative effectiveness of active listening in initial interactions. Int J List. 2014;28(1):13-31. doi:10.1080/10904018.2013.813234

5. Norcross JC, Lambert MJ, editors. Psychotherapy Relationships That Work: Volume 1 — Evidence-Based Therapist Contributions. 3rd ed. Oxford, UK: Oxford University Press; 2019.
