# Supplementary material for: Decision Support Framework for Quality Assurance and Enhancement of Therapeutic Artificial Intelligence Systems: Mixed Methods Pilot Study
Source: JMIR Med Inform. 2026 Jul 23;14:e87887. doi: 10.2196/87887 (PMC13401167; doi:10.2196/87887)
Supplement: Multimedia Appendix 4 [file medinform-v14-e87887-s004.docx]

**Three-Cycle Iterative Enhancement Protocol and Quality Assurance Documentation**

EvaluationPlus Pilot Validation Study — JMIR Medical Informatics Manuscript #87887

## Section A: Three-Cycle Enhancement Protocol

Enhancement recommendations from Stage 2 were implemented across three sequential cycles. Each cycle targeted specific therapeutic competency gaps, with expert clinical review conducted after each cycle before proceeding to the next. Table S1 provides the complete protocol documentation.

**Table S1.** Three-cycle iterative enhancement protocol: modification procedures, targeted competencies, and expert validation outcomes.

| **Cycle** | **Focus** | **Modification Protocol** | **Targeted Competencies** | **Expert Validation Outcome** |
| --- | --- | --- | --- | --- |
| **Cycle 1** | Structural Foundation | Introduced mandatory four-phase structure (Empathy → Exploration → Insight → Action); response-length guidelines imposed | Empathy, Accuracy and Usefulness | Improved consistency; mechanical dialogue observed; excessive literal quotation reduced relational warmth |
| **Cycle 2** | Tone and Flexibility Calibration | Replaced fixed sequencing with flexible scaffolding; severity-adaptive tone modulation; emoji restriction protocol (mild: 🌱😄 → moderate: 😟😰 → crisis: none) | Professionalism, Positivity and Support | Enhanced tone alignment and brevity; personalization still limited |
| **Cycle 3** | Questioning and Natural Personalization | Mandated ≥1 context-relevant follow-up question per response; replaced literal quotation with natural reflective phrasing (e.g., “That sense of being trapped sounds overwhelming”) | Active Listening, Personalization, Complex Thinking | Largest gains: +3.04 (targeted) vs +1.62 (non-targeted); Active Listening +3.20 highest single-dimension gain |

*Note. Each cycle was validated by the supervising clinical psychologist before proceeding. Expert validation used the 3-point clinical rubric described in Multimedia Appendix 1. The emoji protocol specifies: mild scenarios (*🌱😄 *permitted), moderate scenarios (*😟😰 *permitted), crisis scenarios (no emojis).*

## Section B: Design Principles Derived from the Enhancement Process

Three essential principles for therapeutic AI enhancement emerged from the iterative protocol.

**Principle 1: Flexible structural foundation.** Rigid conversational scripts (Cycle 1) reduce naturalness. Flexible scaffolding with severity-based modulation (Cycle 2) preserves therapeutic coherence while enabling contextual adaptation.

**Principle 2: Severity-sensitive communication protocols.** Tone, formality, emoji use, and crisis referral language should be systematically calibrated to user-presented severity, consistent with evidence on therapeutic communication [2,3].

**Principle 3: Expert-guided precision targeting.** Enhancement efforts concentrated on clinically identified deficits (Cycle 3) yield substantially greater gains than diffuse enhancement across all dimensions (targeting differential: +1.42 points).

## Section C: Qualitative Pattern — Mechanical Questioning

Participant feedback from Cycle 3 identified a new conversational pattern: the enhanced system tended to follow each reflective statement with a structured follow-up question, creating a perceivable rhythm of “question + explanation” that some participants described as formulaic. This finding suggests that the mandatory follow-up question rule, while effective in improving Active Listening scores (+3.20), may have introduced a secondary form of conversational rigidity. Future enhancement cycles should explore probabilistic rather than mandatory questioning rules to preserve dialogue naturalness while maintaining therapeutic engagement [2].

## Section D: Quality Assurance Documentation

Structured documentation was maintained throughout the enhancement process: (1) a deficit tracking log mapping each identified gap to its diagnostic source and implemented modification; (2) expert approval records for each modification, including clinical rationale and approval date; (3) version-controlled prompt files with complete change histories; and (4) safety protocol records documenting integration of crisis resource information and escalation language. This QA documentation was adapted from the prior validation framework developed in [1].

**References**

1. Kang B, Hong M. Development and evaluation of a mental health chatbot using ChatGPT 4.0: Mixed methods user experience study with Korean users. JMIR Med Inform. 2025;13:e63538. doi:10.2196/63538. PMID:39705686

2. Hill CE. Helping Skills: Facilitating Exploration, Insight, and Action. 3rd ed. Washington, DC: American Psychological Association; 2009.

3. Weger H, Bell GC, Minei EM, Robinson MC. The relative effectiveness of active listening in initial interactions. Int J List. 2014;28(1):13-31. doi:10.1080/10904018.2013.813234
