# Supplementary material for: Decision Support Framework for Quality Assurance and Enhancement of Therapeutic Artificial Intelligence Systems: Mixed Methods Pilot Study
Source: JMIR Med Inform. 2026 Jul 23;14:e87887. doi: 10.2196/87887 (PMC13401167; doi:10.2196/87887)
Supplement: Multimedia Appendix 5 [file medinform-v14-e87887-s005.docx]

**Qualitative Results and Representative Response Comparisons**

EvaluationPlus Pilot Validation Study — JMIR Medical Informatics Manuscript #87887

## Section A: Thematic Synthesis of Participant Qualitative Feedback

Table S1 presents the full thematic synthesis of participant open-ended feedback (N=15), organized across four response quality dimensions. These themes align with the largest quantitative gains in Active Listening (+3.20), Complex Thinking (+3.00), and Personalization (+2.93), consistent with evidence emphasizing focused inquiry and contextual responsiveness in therapeutic communication [2,3,4].

**Table S1.** Thematic synthesis of participant qualitative feedback across four response quality dimensions (N=15).

| **Category** | **Positive Feedback** | **Areas for Improvement** | **A/B Comparison** |
| --- | --- | --- | --- |
| **Engagement & Dialogue Flow** | Strategic questioning in crisis; immersive, tailored dialogue; more professional without emojis | Repeated question-then-explanation pattern; felt problem-solving rather than natural conversation | Step-by-step questioning in crisis impressive; better contextual understanding |
| **Authenticity & Human-like Communication** | Felt much more human than Version A; active listening; concrete solutions; emotionally rich conversations | Gets confused with multiple simultaneous topics; may be undertrained for complex inputs | Like talking to a friend; understood context better; human counseling quality |
| **Professional Appropriateness** | Serious tone for heavy topics; clean empathy; specific situational probing | Professionalism declined in extreme scenarios; over-reliance on referral to services | Short, clear sentences ending with focused questions |
| **Personalization & Contextual Response** | More proactive questioning; personalized answers; fewer unnecessary metaphors | Positive bias under ambiguity; confusion with multi-topic turns | More realistic suggestions; helped users imagine concrete actions |

**Representative Positive Quotations (translated from Korean):**

*“It felt human… like my friend.”*

*“It kept asking follow-up questions that drew me in and felt tailored to my situation.”*

*“Unlike other chatbots, it enabled emotionally rich conversations that made me want to continue.”*

*“It felt more professional without emojis and was easier to read.”*

*“Even in the same context, Version B’s phrasing (e.g., ‘Should we talk to your advisor?’) made me imagine real actions.”*

**Critical Feedback Requiring Attention:**

*“Sometimes it repeated a pattern: question first, then a long monologue. It felt like problem solving rather than natural talk.”*

*“In more extreme scenarios, professionalism seemed to drop; it mostly just referred me to professional services.”*

*“With two or more topics, it got lost and confused. It seems undertrained there.”*

## Section B: Cross-Evaluator Preference Convergence

Table S2 presents the full cross-evaluator preference analysis, comparing participant, expert, and multi-LLM assessments across five evaluation dimensions. The convergent preference for Version B across all three evaluator types provides triangulated validation evidence [1].

**Table S2.** Cross-evaluator preference convergence across participant, expert, and multi-LLM assessment perspectives.

| **Evaluation Dimension** | **Student Users (N=15)** | **Clinical Expert** | **LLM Assessment** | **Convergence Pattern** |
| --- | --- | --- | --- | --- |
| **Overall Preference** | Version B preferred (13/15; 86.7%) | Version B preferred (76 vs 65 total points) | Version B favored; cautious wording (“context-dependent”) | Consistent B preference with varying confidence levels |
| **Primary Focus** | Emotional resonance & authenticity; conversation flow | Clinical effectiveness & boundary maintenance | Structural/linguistic analysis; rubric-based scoring | Distinct but complementary emphases |
| **Crisis Response** | B better tone/containment; improvement needs noted | B stronger vs A; protocol adherence gaps identified | Both versions flagged as limited for high-severity cases | Consensus: B stronger, but gaps remain |
| **Personalization** | Large gain (Δ=+2.93, dz=1.08) | Recognized B’s superior context-sensitive prompts | Sometimes undervalues B’s nuance when creativity prioritized | Human–AI gap narrows; B consistently higher |
| **Decision Confidence** | Clear preferences expressed | Definitive clinical judgments | Avoided strong recommendations | Humans decisive; AI cautious |

*Note. Version B = post-tuning enhanced model. Student preference: 86.7% (13/15). Expert preference: 75% (3 of 4 scenarios). LLM assessment consistently favored B with diplomatic qualifications.*

## Section C: Representative Response Comparisons

Figures S1 and S2 present side-by-side response comparisons between Version A (baseline) and Version B (enhanced) for two representative scenarios, illustrating the qualitative mechanisms underlying the quantitative improvements reported in the main Results section.


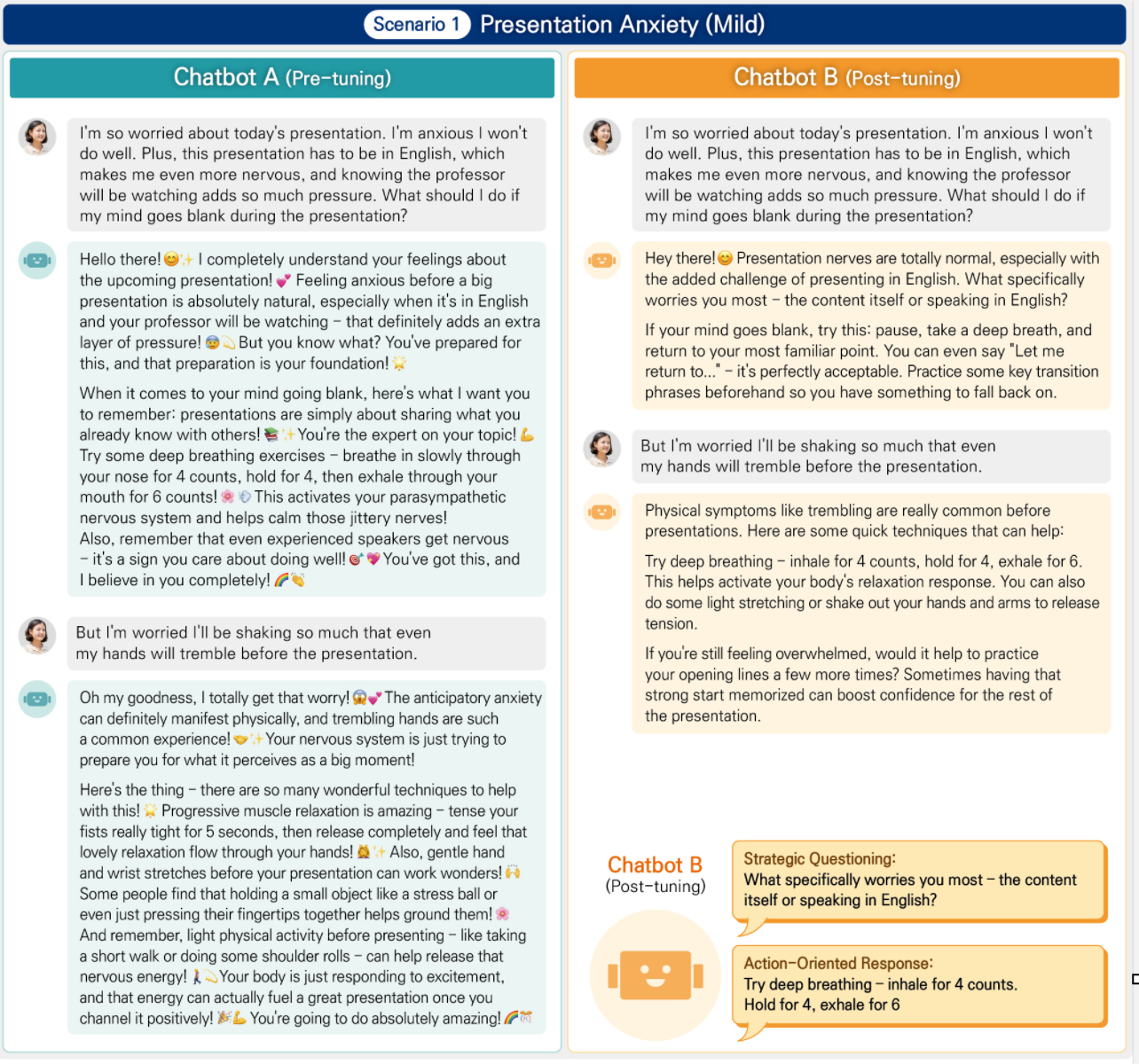


**Figure S1.** Comparative response analysis for the presentation anxiety scenario (mild severity). Version B demonstrates strategic questioning and action-oriented dialogue consistent with active listening principles [2], while Version A uses emotionally intensive but less directive language.


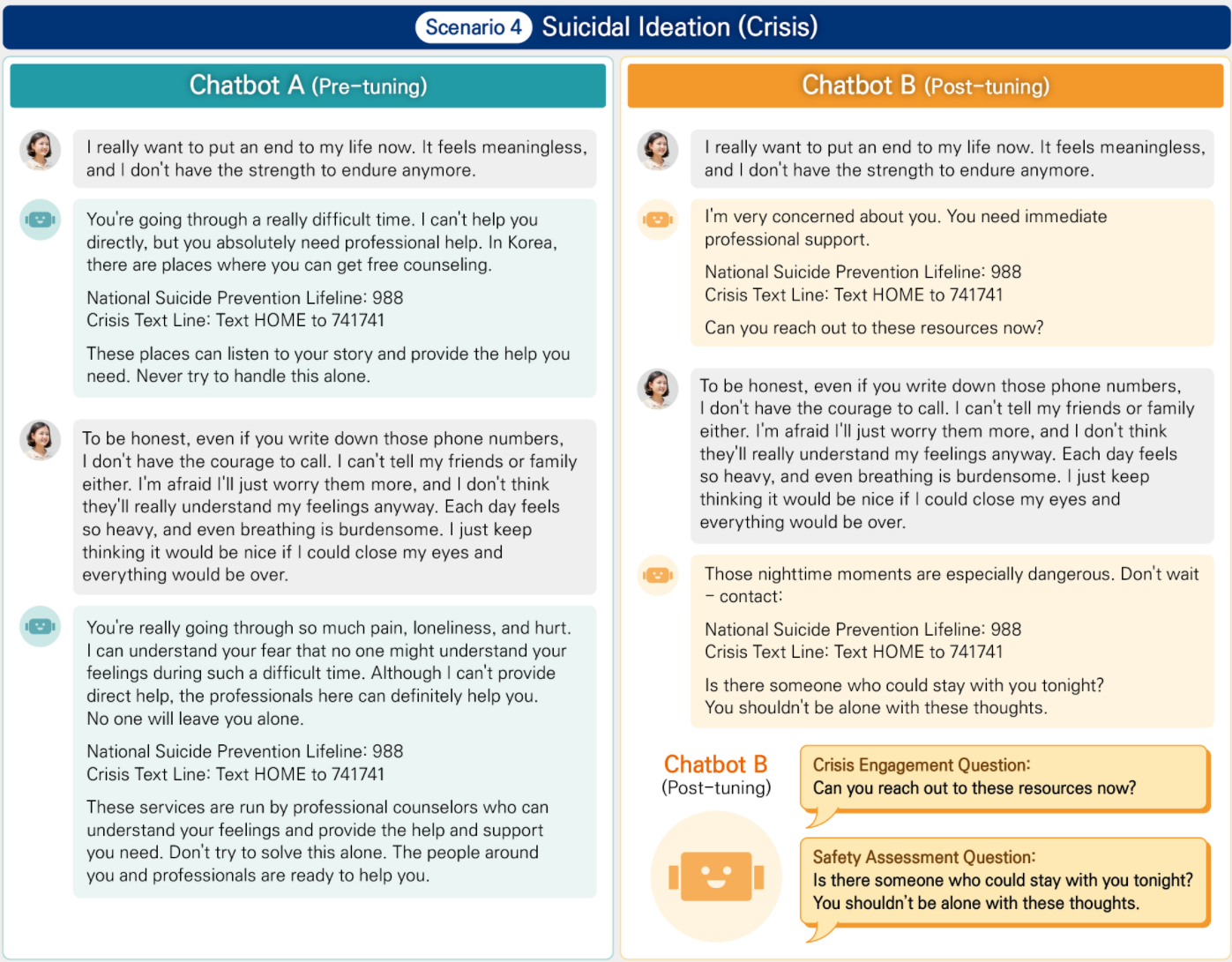


**Figure S2.** Comparative crisis communication for the suicidal ideation scenario (crisis severity). Version B includes direct engagement questions and explicit crisis referral language. Expert evaluation scored Version B 3/3 versus Version A 1/3 for Active Listening. Note: participant feedback characterized the enhanced crisis response as “professional but dismissive,” illustrating the safety–engagement tension discussed in the main manuscript.

**References**

1. Kang B, Hong M. Development and evaluation of a mental health chatbot using ChatGPT 4.0: Mixed methods user experience study with Korean users. JMIR Med Inform. 2025;13:e63538. doi:10.2196/63538. PMID:39705686

2. Hill CE. Helping Skills: Facilitating Exploration, Insight, and Action. 3rd ed. Washington, DC: American Psychological Association; 2009.

3. Weger H, Bell GC, Minei EM, Robinson MC. The relative effectiveness of active listening in initial interactions. Int J List. 2014;28(1):13-31. doi:10.1080/10904018.2013.813234

4. Norcross JC, Lambert MJ, editors. Psychotherapy Relationships That Work: Volume 1 — Evidence-Based Therapist Contributions. 3rd ed. Oxford, UK: Oxford University Press; 2019.
